# Health policy and systems research for rehabilitation: a call for papers

**DOI:** 10.2471/BLT.21.287200

**Published:** 2021-10-01

**Authors:** Alarcos Cieza, Aku Kwamie, Qhayiya Magaqa, Abdul Ghaffar

**Affiliations:** a Sensory Functions, Disability and Rehabilitation Unit, Department of Noncommunicable Diseases, World Health Organization, Avenue Appia 20, 1211 Geneva 27, Switzerland.; b Alliance for Health Policy and Systems Research, World Health Organization, Geneva, Switzerland.

Rehabilitation is included in the universal health coverage (UHC) target of the sustainable development goals as an essential health service; access to rehabilitation is a human right.^1^ Rehabilitation services are interventions required when people have limitations in their daily physical, mental and social functioning due to ageing or health conditions, including chronic diseases or disorders, injuries or trauma.^2^ The ultimate objective of rehabilitation is to enable the person to achieve their optimal level of functioning in their environment. The coronavirus disease 2019 (COVID-19) pandemic is a reminder that rehabilitation may also be necessary in the acute, post-acute and long-term phases of infectious diseases.^3^^,^^4^

Current estimates report that 2.4 billion people have health conditions that lead to limitations in functioning severe enough for them to need rehabilitation services.^5^ This figure is expected to rise as people live longer, and with a higher number of chronic diseases.^2^ Hence the necessity to position rehabilitation as an important global health priority. Such prioritization is relevant given that the need for rehabilitation services is largely unmet. In some low- and middle-income countries, for example, most people do not receive the rehabilitation services they require.^6^

The post COVID-19 condition provides the world a glimpse of what it means to live with limitations in functioning and how fundamental functioning is to people’s well-being. Not being able to move around, think clearly or work are common limitations in functioning associated with health conditions that benefit from rehabilitation. This lesson from COVID-19 provides an opportunity for the global health community to recognize that functioning is the third health indicator alongside mortality and morbidity.^7^ Governments must also recognize that rehabilitation is the health systems’ strategy that directly contributes to improved functioning, and thus to well-being. Overcoming the tendency of the health sector to primarily focus on prevention and treatment and to exclude rehabilitation and the needs of people with limitations in functioning is timely. The use and application of robust health policy and systems research will deliver the necessary evidence to equip health systems to be responsive to the needs for rehabilitation.^8^

Situating rehabilitation as a central tenet of health systems rather than a vertical programme in health or other sectors enables a more holistic appreciation of the integrative nature of rehabilitation. Rehabilitation services can be delivered by specialized personnel in tertiary facilities. However, rehabilitation services should also be integrated at secondary, primary and community level as well as across other health services. 

Political commitment is essential to mobilize resources for rehabilitation and to increase the demand for rehabilitation services. Health policy and systems research can generate evidence for informing policy and practice decisions.^9^ In this context, such research seeks to understand and improve how societies organize themselves in achieving the collective health goal of optimizing functioning and the role that rehabilitation plays in health systems towards that end. This research also analyses how different actors interact in the policy and implementation processes to contribute to policy outcomes,^10^ such as the political prioritization of rehabilitation.

Health policy and systems research can, for example, provide evidence on how countries can reorganize their health systems to deliver rehabilitation services for their populations. Such research lends itself to the emerging questions for rehabilitation in the 21st century and is fit for purpose for gathering evidence to implement the World Health Organization’s Rehabilitation 2030 initiative with its 10 areas of action.^11^ This initiative marks a new strategic approach for the global rehabilitation community by emphasizing that efforts to strengthen rehabilitation should be directed towards supporting the health system as a whole and integrating rehabilitation into all levels of health care.

The *Bulletin of the World Health Organization* will publish a theme issue on advancing rehabilitation through health policy and systems research. We welcome papers that expand our current knowledge by presenting rehabilitation as a relevant strategy in health, including public health, that needs to be scaled up to adequately address population needs. Papers may focus on strategies and service provision models and on options for rehabilitation in health financing as a part of each country’s journey towards UHC. Successful and sustainable health systems models for integrating rehabilitation into primary care, approaches to community mobilization for increasing the demand for rehabilitation or models for increasing service delivery by strengthening the workforce are also of interest. The deadline for submission is 28 February 2022. Manuscripts should be submitted in accordance with the *Bulletin’s* guidelines for contributors (available at: https://www.who.int/publications/journals/bulletin/contributors/guidelines-for-contributors) and the cover letter should mention this call for papers.
